# Clinical Investigation of the Inhibitory Effects of Tooth-Coating Materials on Initial Active Root Caries: A Pilot Randomized Controlled Trial

**DOI:** 10.3390/medicina60010150

**Published:** 2024-01-13

**Authors:** Yoko Asahi, Katsuaki Naito, Hikaru Kanda, Kazuaki Niwano, Daisuke Takegawa, Hiromichi Yumoto, Yuichiro Noiri, Mikako Hayashi

**Affiliations:** 1 Department of Restorative Dentistry and Endodontology, Osaka University Graduate School of Dentistry, Osaka 565-0871, Japan; naito.katsuaki.dent@osaka-u.ac.jp (K.N.); kanda-hikaru@dent.osaka-u.ac.jp (H.K.); hayashi.mikako.dent@osaka-u.ac.jp (M.H.); 2 Division of Cariology, Operative Dentistry and Endodontics, Department of Oral Health Science, Niigata University Graduate School of Medical and Dental Sciences, Niigata 951-8514, Japan; niwano@dent.niigata-u.ac.jp (K.N.); noiri@dent.niigata-u.ac.jp (Y.N.); 3 Department of Regenerative Dental Medicine, Tokushima University Graduate School of Biomedical Sciences, Tokushima 770-8504, Japan; d-takegawa@tokushima-u.ac.jp; 4 Department of Periodontology and Endodontology, Tokushima University Graduate School of Biomedical Sciences, Tokushima 770-8504, Japan; yumoto@tokushima-u.ac.jp

**Keywords:** root caries, secondary prevention, glass ionomer cement, Caredyne ZIF-C, biofilm

## Abstract

*Background and Objectives*: Caredyne ZIF-C is a novel, capsule-mixed zinc-containing prototype glass ionomer cement (GIC). Zinc ions are reported to inhibit root dentin demineralization, dentin collagen degradation, bacterial growth, acid production, and in vitro bacterial biofilm formation. However, the effectiveness of GICs against initial root caries lesions is unclear. Therefore, this study aimed to evaluate the efficacy of GICs, especially the new zinc-containing Caredyne ZIF-C GIC, as tooth-coating materials in patients with initial active root caries. *Materials and Methods*: A total of 58 lesions in 47 older adults (age > 65 years) were randomly allocated to one of the following three groups: Caredyne ZIF-C, Fuji VII (a conventional GIC), and sodium fluoride (NaF). All the lesions were treated with the assigned materials without removing the infected dentin, and the rates of dental plaque attachment and coating material fall-out were evaluated after 3, 6, and 12 months. The failure rate was defined as the number of teeth that needed restoration due to caries progression. *Results*: The plaque attachment rates tended to be lower in the material-coated root surfaces than in the healthy exposed root surfaces after 3, 6, and 12 months, although the differences among the three groups were not significant. Moreover, the coating material fall-out rate tended to be lower in the Caredyne ZIF-C group than in the Fuji VII group. There was no significant difference in the failure rate among the three groups at the 12 months mark. *Conclusions*: Though this pilot study offers a new direction for suppressing the progression of initial active root caries by controlling plaque attachment using GICs including Caredyne ZIF-C, clinical studies with a larger sample size are needed.

## 1. Introduction

The worldwide increase in life expectancy and advances in dental care have led to the increased retention of teeth in older individuals. However, the prevalence of gingival recession increases with age; therefore, the incidence and prevalence of root caries in this population have also increased, currently posing a significant problem worldwide [1,2]. Root surface dentin has a critical pH of <6.4, which is higher than that of enamel (<5.5), as well as a lower mineral content than that of enamel, making it more susceptible to caries [3]. Additionally, it is difficult to determine the range of repair treatment for initial root caries because the margins of the lesion are unclear, and the removal or repair of the lesion is challenging in many cases. Moreover, teeth damaged by root caries often need to be extracted, resulting in reduced oral function. Therefore, demineralization and formation of new root caries lesions should be prevented by improving the acid resistance of root surfaces susceptible to caries.

For initial demineralized lesions, a non-invasive treatment to inhibit caries progression and achieve the remineralization of the infected root dentin without “drilling and filling” is considered more beneficial for patients [4,5]. Several approaches have been proposed for inactivating root caries lesions, most of which involve the use of fluoride. In the literature review by Gluzman et al. [6], the recommendations for secondary prevention of root caries in the general adult population and vulnerable older individuals are the daily usage of a 4500–5000 ppm sodium fluoride (NaF) toothpaste gel as self-care and the application of 22,500 ppm NaF varnish every 1–3 months as professional care. According to the meta-analysis by Wierichs et al., dentifrices containing 5000 ppm fluoride or 1450 ppm fluoride with 1.5% arginine were more effective at inactivating root caries lesions (inactivating 51% more root caries lesions with 5000 ppm fluoride and 21% with 1450 ppm fluoride with 1.5% arginine) than dentifrices containing 1100–1450 ppm fluoride [7]. Another meta-analysis reported that, compared with no treatment, the use of a 38% silver diamine fluoride solution alone or with potassium iodide applied annually, 5% NaF varnish, or 1% chlorhexidine with 1% thymol varnish was associated with a two to three times greater chance of arresting or reversing root surface lesions, although the certainty of the evidence was very low [8].

In contrast, only few clinical studies have investigated the efficacy of tooth surface-coating materials in the secondary prevention of the demineralization of root caries lesions, although the dentin adhesives used as sealants in these studies have shown promising results in preventing further demineralization [9,10]. A few in vitro studies investigating the efficacy of dentin sealants in preventing root caries have claimed that dentin bonding can stabilize caries-affected areas by protecting the exposed surfaces and preventing the progression of demineralization [11,12]. Though bonding sealant acts as only a physical barrier shielding the lesion from further acid attacks, it could serve as an alternative approach for patients with initial root caries who have difficulty in receiving frequent professional care involving fluoride application or self-care with high-concentration fluoride dentifrices.

Glass ionomer cements (GICs) have been used as restorative materials. GICs are commonly used in the treatment of advanced root caries [13,14], as these have proven useful in cases of the extension of caries to the subgingival area or when moisture control is difficult [4]. GICs adhere to the tooth surface directly and release fluoride, which inhibits bacterial growth and enhances tooth structure by forming fluorapatite [15]. Remineralization can occur owing to changes in environmental conditions that spread within the dental biofilm-covered carious lesions [16]. Thus, controlling and inhibiting dental biofilm formation or the application of materials that have an effect on hardening dentine could inhibit demineralization and promote remineralization. An in vitro study using a bovine carious dentin model showed that both conventional GIC and a mineral trioxide aggregate induced mineral density gain [17]. In previous clinical studies, GIC sealants were shown to be effective in reducing the progression of initial enamel proximal caries [18] or arresting microcavitated (ICDAS 3) coronal carious lesions [19]. Therefore, the application of GICs as a coating material is expected to inhibit dental biofilm formation and surface root caries progression, and its effectiveness is expected to be higher than that of resin bonding, which is another candidate coating material which has no antibacterial or remineralization effects.

Caredyne ZIF-C (GC Dental Industrial Corporation, Tokyo, Japan) is a novel capsule-mixed zinc-containing prototype GIC, which is a commercially available variant of GIC Caredyne Restore; it contains a BioUnion filler [20] and releases calcium, fluoride, and zinc ions. Reportedly, zinc ions can inhibit root dentin demineralization, dentin collagen degradation, bacterial growth, acid production, and biofilm formation in vitro [21,22,23,24,25,26,27]. Using GICs as coating materials for initial active root caries not only physically protects carious lesions but is also expected to suppress root caries progression through their anti-biofilm effects and improvement of dentine structure through the incorporation of fluoride. Caredyne ZIF-C would have an especially strong effect as it contains zinc in addition to fluoride. However, to the best of our knowledge, no clinical study has reported on the use of GICs as coating materials for the management of initial root caries; therefore, the effect of GICs against plaque attachment, caries progression, or remineralization of initial root caries lesions is unclear. Thus, our research objective was to assess the extent of stabilization of root caries lesions with GICs as the tooth-coating materials. Focusing on the regulation of biofilm formation, the aim of this study was to evaluate the effects of Caredyne ZIF-C and a conventional GIC (Fuji VII Capsule; GC Dental Industrial Corporation, Tokyo, Japan) on dental biofilm attachment on initial active root caries, in comparison to a NaF solution. Moreover, we assessed the failure rate, which was defined as the need for restoration due to caries progression. The NaF solution was selected as the target for comparison because it is commonly used in Japan. We hypothesized that Caredyne ZIF-C, which contains zinc in addition to fluoride and inhibits biofilm formation more significantly than Fuji VII in vitro [21], would have the highest plaque attachment inhibitory effect; we conducted a pilot randomized controlled trial that included 18 teeth in each group.

## 2. Materials and Methods

### 2.1. Study Design

This pilot randomized controlled trial was conducted at three universities in Japan using a parallel-group design. The study protocol was reviewed and approved by the Ethics Committee of the Osaka University Graduate School of Dentistry (H30-E40-3), the Ethics Committee of Niigata University (Approval number 2019-0007), and the Ethics Committee of Tokushima University Hospital (3573-3) and was registered prior to participant recruitment with the University Hospital Medical Information Network-Clinical Trial Registry (UMIN-CTR; No. UMIN000035609, registered on 22 January 2019).

### 2.2. Recruitment

Older patients (age > 65 years) were recruited at three university hospitals: Osaka University Dental Hospital, Niigata University Medical and Dental Hospital, and Tokushima University Hospital. The inclusion criterion was the presence of at least one permanent tooth with initial active root caries lesions, specifically those classified as code 1 of the International Caries Detection and Assessment System (ICDAS II) classification [28] and soft/leathery lesions. Patients with advanced root caries that needed drilling and those who could not visit the hospital every 3 months were excluded from this study. The informed consent form was explained to and read by the patients, and written informed consent was obtained from all the participants prior to clinical examinations.

### 2.3. Baseline Examinations

Clinical examinations were performed, and information regarding the number of remaining teeth, the decayed, missing, and filled teeth score, and the plaque control record was obtained. The root caries lesions were also assessed by means of a visual and tactile examination to evaluate the color (yellow/light brown/dark brown/black) and hardness (soft/leathery). Saliva was stimulated using a gum provided in the kit and was collected to calculate the salivary flow rate and the salivary bacterial counts. The Saliva-Check LAB^®^ (GC Dental Industrial Corporation, Tokyo, Japan) was used according to the manufacturer’s instructions to estimate streptococci, mutans streptococci, and lactobacilli counts using a real-time polymerase chain reaction, as previously reported [29].

### 2.4. Randomized Allocation

An independent researcher performed randomization using a random number table. The original random allocation sequence was maintained at a separate location. The patients were randomly allocated in a double-blind manner to one of the following three treatment groups: Caredyne, FUJI VII, and NaF. If a patient had multiple teeth with root caries lesions, more than two teeth per patient were included in the study; however, the same treatment method was applied to all teeth in an oral cavity.

### 2.5. Interventions

The tooth-coating materials used in this study (two sealants and one fluoride solution) are shown in Table 1.

The target teeth were professionally cleaned and isolated using cotton rolls; the saliva was removed using a cotton pellet, and the teeth were dried using a three-way syringe. The allocated material was then applied without removing the carious dentin. The fluoride solution was applied for 4 min. For the sealants, the tooth surface was first treated with a cavity conditioner for 10 s and then washed and dried. The sealants were mechanically mixed for 10 s (Capsule Mixer CM-II; GC Dental Industrial Corporation, Tokyo, Japan), applied to the root surface, and coated with varnish.

### 2.6. Follow-Up Examinations

Follow-up examinations were conducted at 3, 6, and 12 months after the baseline by the same examiner. The color and texture of the lesions and the adhesion of the sealants were evaluated via an inspection. The hardness of the lesions was estimated by means of palpation using a Community Periodontal Index (CPI) probe [30,31]. The color and hardness of the lesions and the degree of demineralization in the GIC groups were evaluated to the extent possible, even in cases wherein the coating material was detached. The rate of attachment of dental plaque was assessed based on oral photographs obtained after staining the plaque with Butler GUM RED-COTE (Sunstar, Osaka, Japan). The root surface area and plaque attachment area on the oral photographs were measured using a polygon selections tool in an image analysis software (ImageJ version 1.53; NIH, Bethesda, MD, USA), and the residual rate of plaque on the root surface was calculated. Healthy exposed root surfaces in each participant were used as the control. We selected the same type of tooth as the coated tooth to the extent possible in the following priority order: adjacent tooth, opposite tooth, and opposing tooth. The image analysis was performed by an independent researcher who was blinded to the treatment groups, and the data were encoded. The failure rate was calculated by the number of teeth that needed restoration due to caries progression. The fluoride solution in the NaF group was applied every 3 months, whereas the GIC sealants in the Caredyne ZIF-C and Fuji VII groups were re-applied only if a partial or complete loss of the sealant was noted. Calibration was performed before the start of the trial to ensure the uniformity of the assessment methods and techniques.

### 2.7. Sample Size

No previous clinical studies were available for reference regarding sample size calculation; therefore, a minimum of 15 teeth were estimated to be required in each group [32]. Assuming a 15% dropout rate, 18 teeth were included in each group in this study.

### 2.8. Statistical Analysis

All statistical analyses were performed using SPSS ver. 22.0 (IBM SPSS Inc., Armonk, NY, USA). This study was conducted under the intention-to-treat principle. Fisher’s exact test was used to analyze the sealant material fall-out rate, the failure rate, the baseline characteristics of the teeth, and potential sex differences. The Kruskal–Wallis test was used to assess the residual rate of dental plaque and compare the patient characteristics at the baseline. Spearman’s rank correlation coefficient analysis was used to assess the correlation between salivary flow and dental plaque accumulation. The level of statistical significance was set to 5%.

## 3. Results

**Figure 1:**
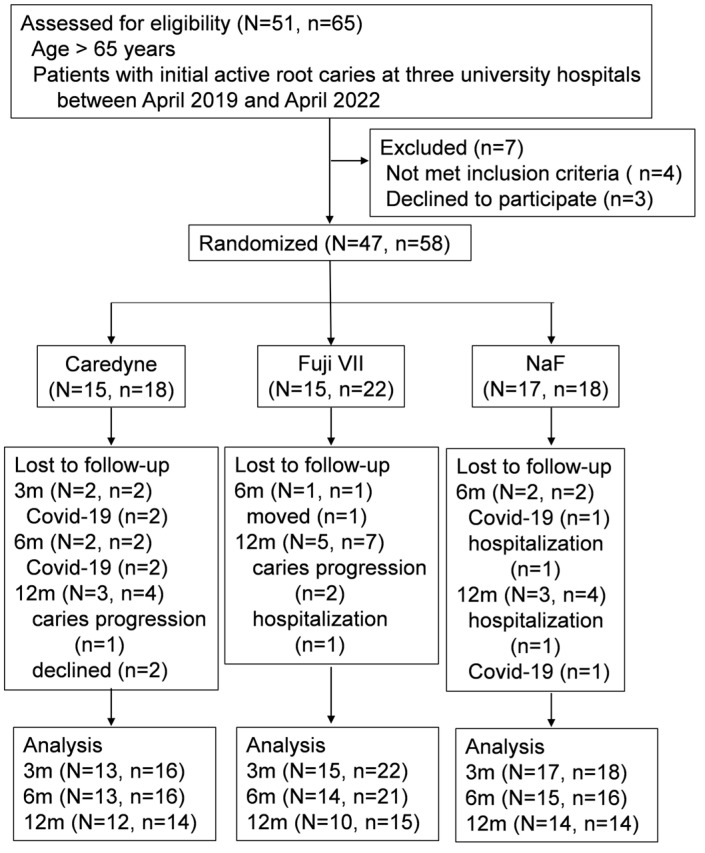
Flowchart of the study participants. N = participants with root caries, n = root caries lesions.

Fifty-one older adults (65 teeth) underwent dental examinations at the three university hospitals between April 2019 and April 2022, and 47 individuals (58 teeth) who met the eligibility criteria and agreed to participate were enrolled in this study. After 3, 6, and 12 months, the dropout rates were, respectively, as follows: 11.1%, 11.1%, and 22% in the Caredyne ZIF-C group; 0%, 4.5%, and 31.8% in the Fuji VII group; and 0%, 11.1%, and 22% in the NaF group (Figure 1).

The main reasons for dropout were related to hospitalization and COVID-19.

Table 2 presents the baseline demographic characteristics of the study participants; no statistically significant differences were observed among the three groups in terms of any of these variables.

Table 3 shows the distribution of tooth types and affected sites, which were scattered among the three groups.

The baseline characteristics of the root caries lesions are presented in Table 4; no statistically significant differences in color, texture, or hardness were observed.

**Figure 2:**
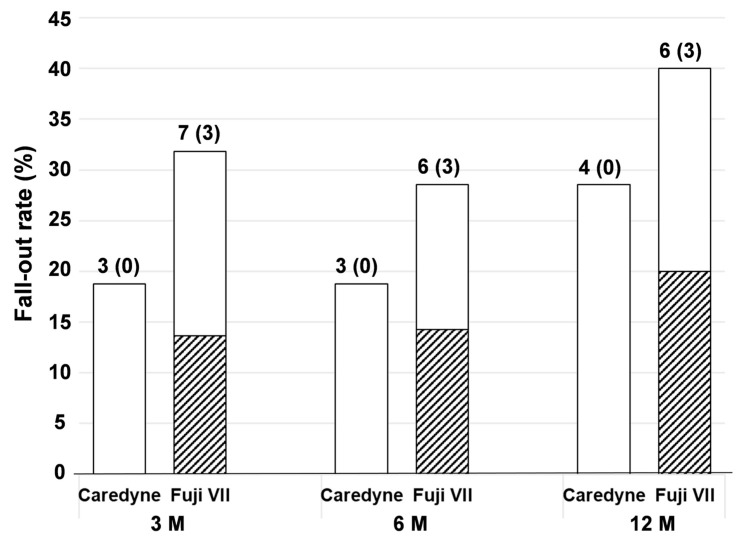
Fall-out rate of the coating materials. The hatched bar indicates partial defects. The numbers above the bar indicate the sample size. Parentheses indicate partial defects. Fisher’s exact test, *p* > 0.05.

Figure 2 shows the fall-out rates of the Caredyne ZIF-C and Fuji VII coating materials.

Although the material fall-out rate tended to be higher in the Fuji VII group, the difference was not significant. The rate of partial loss of the material was higher in the Fuji VII group than in the Caredyne ZIF-C group. The fall-out of material was not found to be associated with lesions’ surface hardness or tooth type (Table S1). The detachment of materials occurred more frequently on the defect side of the clasped tooth. Among the lesions with fall-out, some had improved lesion texture, from rough to smooth, and lesion hardness, from soft/leathery to hard, while others showed caries progression and needed drilling and filling.

**Figure 3:**
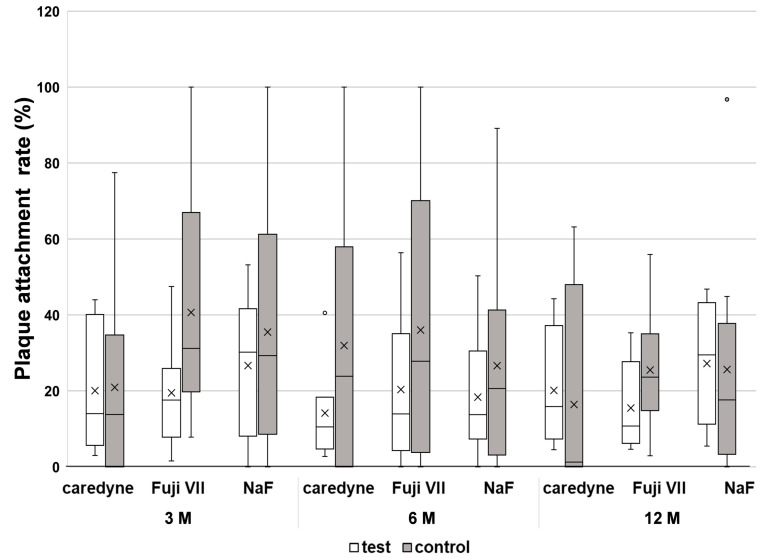
Rate of dental plaque attachment. “Test” indicates material-coated root surfaces, and “control” indicates healthy exposed root surfaces. Circles represent outliers. “X” indicates the mean. Kruskal–Wallis test, *p* > 0.05.

The outcomes related to the attachment rate of dental plaque are presented in Figure 3.

Although the Kruskal–Wallis test did not reveal any significant differences among the three groups in the rate of dental plaque attachment at 3, 6, and 12 months, the rates tended to decrease in the treatment groups. There were no strong correlations between the salivary flow rate and the rate of dental plaque attachment (3 months: *rs =* −0.027; 6 months: *rs =* −0.104; and 12 months: *rs =* −0.41).

Between 3 and 6 months after the baseline, one patient in each of the GIC groups required restorative treatment due to the progression of root caries, resulting in failure rates of 5.88% in the Caredyne ZIF-C group and 4.65% in the FUJI VII group (*p* = 0.621). The treated teeth in both the Caredyne ZIF-C and Fuji VII groups were clasped teeth on the defect side. Between 6 and 12 months after the baseline, one patient in each group required restorative treatment due to the progression of root caries, and the failure rates were 6.25% in the Caredyne ZIF-C group, 5.4% in the FUJI VII group, and 6.25% in the NaF group (*p* = 0.998).

## 4. Discussion

Currently, high-concentration fluoride liquids or varnishes are used in professional care to effectively arrest root caries lesions. In this study, we aimed to stabilize the lesions by suppressing the progression of root caries as well as by physically protecting them from acid challenges in the long term. In this pilot study, the following results were obtained. There were no significant differences in the failure rates among the three groups at 12 months. Although the plaque attachment rates tended to be lower in the GICs-coated root surfaces than in the healthy exposed root surfaces, there were no significant differences. Moreover, the coating material fall-out rate tended to be lower in the Caredyne ZIF-C group than in the Fuji VII group. Although no significant differences were obtained, indicating a lack of immediate clinical application, this study demonstrated the potential for managing initial active root caries. Consequently, further clinical research in this area is warranted. While fluoride application is widely used in the management of initial root caries, the results of this study might lead to a new option for secondary prevention, which involves coating root caries lesions with bioactive materials.

The fall-out rates for the GIC coatings used in this study were higher than those in the daily treatment. In previous works, demineralization was reported to have a significant effect on the dentin bond strengths of Caredyne ZIF-C and Fuji VII, though these bond strengths were less influenced when compared to those of resin composites [33]; coating root caries lesions without the removal of carious dentin may induce a higher fall-out rate. In a study by Wicht et al., root caries lesions were sealed after the removal of the infected dentin [9], whereas, in another study by Baysan et al., root sealant was applied to the lesions without removing the carious dentin [10]. The removal of carious dentin prior to coating a lesion is expected to reduce the fall-out rates of materials, although it has not yet been established whether infected dentin should be removed or not.

The fall-out rates were approximately 20% (Caredyne ZIF-C group) and 30% (Fuji VII group) in this study after 3 months and 6 months and increased to 30% (Caredyne ZIF-C group) and 40% (Fuji VII group) after 12 months. Therefore, the recall period for a GIC-coated tooth can be considered to be 6–12 months. Hence, compared to the established fluoride treatments, the assumed interval of intervention will be longer in the GICs coating approach. Meanwhile, since there were no significant differences in the failure rate (the rate of teeth which needed restoration) after 12 months, it is indicative that coating with GICs is as cost-effective as coating with fluoride. In particular, the protection of lesions with GICs might have a potential advantage over fluoride application for patients who have difficulty in frequently visiting a dental clinic or engaging in self-care. The defect side of the clasped tooth appeared to have some difficulty retaining the coating materials; therefore, it is recommended to avoid using them on clasped teeth.

Partial defects of the coating material were observed in the Fuji VII group. Nagasawa et al. investigated the effects of saliva contamination and/or dentin demineralization on the shear bond strength between bovine root dentin and Caredyne ZIF-C, Fuji VII, and resin composites [33]. They reported that cohesive failure was common with Fuji VII regardless of the experimental conditions, although adhesive failure was also observed with Caredyne ZIF-C, especially under demineralized dentin without saliva and with severe saliva contamination. They also reported that the compressive strength, diametral tensile strength, and flexural strength of Fuji VII were lower than those of other restorative materials and that Caredyne ZIF-C was more difficult to wear and disintegrate than Fuji VII and fell out in blocks, without chipping.

We have previously reported that Caredyne Restore (GC Dental Industrial Corporation, Tokyo, Japan), a non-capsule-type Caredyne ZIF-C variant, inhibited *Streptococcus mutans* biofilm formation in vitro by interfering with bacterial adhesion more effectively than hydroxyapatite disks and FUJI VII (*p* < 0.05) [21]. Similarly, Nishida et al. reported that zinc-fluoride glass nanoparticles (Zinc-F) suppressed *S. mutans* and *Actinomyces naeslundii* biofilm formation; they hypothesized that Zinc-F had anti-biofilm effects and inhibited the growth of oral bacteria by releasing multiple ions [34]. In the present study, although the dental plaque attachment rate showed a downward trend, a significant difference was not observed, unlike what had been expected. While the above in vitro studies evaluated anti-biofilm effects after 24 h of incubation, we evaluated the residual dental biofilm 3 months after professional cleaning, and biofilm maturation in this context might be different from that observed in vitro, which may have led to non-significant results. To investigate the effects of tooth-coating materials on bacterial adhesion, it is necessary to evaluate them over a shorter period of time. Notably, Caredyne Restore was found to reduce biofilm thickness and the ratio of live/dead bacteria in vitro [35]. It would be interesting to clarify the multi-faceted effects of these tooth-coating materials on oral cavity biofilms. Another potential reason for the non-significant results is the large variation in plaque attachment rate in the controls (healthy exposed root surface) among the three groups. Therefore, we will consider comparing the plaque attachment at the baseline as well as after coating the materials on the same root surface in the future.

Kohno et al. reported that the concentration of Zn^2+^ released from a GIC-containing BioUnion filler under acidic conditions over 7 days could be maintained at the level required to hinder *S. mutans* and multi-species biofilm formation in vitro [35]. However, F^-^ release from both the GIC-containing BioUnion filler and Fuji VII gradually decreased over 7 days, and its concentrations, although higher under acidic conditions, were below the level required to inhibit *S. mutans* and multi-species biofilm formation in vitro. Furthermore, they indicated that the GIC used in this study could recharge the Zn^2+^ and F^-^ in the BioUnion filler. Although there were no significant differences in the results obtained in this study, considering that Caredyne ZIF-C is expected to have a sustained anti-biofilm effect due to Zn^2+^ in addition to F- and that its fall-out rate tended to be lower than that of Fuji VII, Caredyne ZIF-C might be more suitable than Fuji VII as a tooth-coating material for initial active root caries. We look forward to the results from future large-scale clinical studies.

This study has some limitations, including its small sample size. Taking the results of this study into consideration, the calculated sample size should be around 140 subjects per group. Future research should include large multi-center prospective studies. Another limitation was the fact that changes in cement-coated tooth surfaces were difficult to evaluate unless the tooth-coating materials were removed. Therefore, the root caries preventive rate or caries arrest rate could not be evaluated, and the only parameter that could be evaluated regarding caries progression was the failure rate, defined as the need for restoration due to caries progression. There were no significant differences in failure rates among the groups, suggesting that root caries stabilization among the materials may be comparable. However, one or two teeth in each group needed restoration, and measuring dentin hardness may have allowed for a more detailed evaluation of the differences among the materials. Therefore, we are considering an evaluation of the possibility of removing the cement without damaging the caries lesion. Additionally, it is desirable to evaluate hardness in future research.

Based on the results of this pilot study, to verify the potential of GICs as coating materials against initial active root caries, it is necessary to improve the evaluation method for lesion surface hardness. Additionally, it is necessary to reevaluate the timing of evaluation and determine the appropriate control for plaque attachment, as stated earlier. Further research on this aspect is warranted.

## 5. Conclusions

Although no significant differences were noted in the failure rate and plaque attachment rate among GICs coating and fluoride application, several areas for improvement in the methods were noted for future research. Thus, this pilot study is the first step of a clinical study using GICs as the tooth-coating materials for initial active root caries, and it forms the foundation of the main study. Although fluoride application is the gold standard in the field of root caries management, this pilot study may offer a new direction in the management of initial active root caries, in which additional effects by coating can be expected in addition to the anti-carious effects of GICs. Caredyne ZIF-C can especially be expected to have anti-biofilm and anti-demineralization effects not only due to F^-^ but also due to Zn^2+^. Although there was no significant difference, the fall-out rate tended to be lower than that of Fuji VII; therefore, it might be more desirable as a tooth-coating material for the secondary prevention of root caries. As the next steps in research, we are planning a study to assess the possibility of removing cement materials without damaging the tooth surface to evaluate the latter’s hardness. Further, we are planning to conduct a larger clinical trial that employs the contemplated approach for quantifying tooth hardness, along with enhanced methodologies for assessing the rate of plaque attachment.

## Figures and Tables

**Table 1:** Tooth-coating materials used in this study.

Material	Composition	Manufacturer
Caredyne ZIF-C	Powder: fluoroaluminosilicate glass, fluorozincsilicate glass (BioUnion filler), polyacrylic acid Liquid: polyacrylic acid, distilled water, polybasic carboxylic acid, phosphoric acid	GC Dental Industrial Corporation, Tokyo, Japan
FUJI VII capsule	Powder: fluoroaluminosilicate glass Liquid: polyacrylic acid, distilled water, polybasic carboxylic acid	GC Dental Industrial Corporation, Tokyo, Japan
Sodium fluoride solution 2%	2% acidulated phosphate sodium fluoride	BEE BRAND MEDICO DENTAL. CO., LTD, Osaka, Japan

**Table 2:** Baseline demographic characteristics of the participants.

	Caredyne	Fuji VII	NaF	*p*-Value
Mean age (SD)	72 (5.5)	75 (6.2)	73 (6.4)	0.26
Sex				0.13
Male	7 (47%)	9 (60%)	4 (23.5%)	
Female	8 (53%)	6 (40%)	13 (76.5%)	
Mean number of remaining teeth (range)	24 (19–29)	20 (4–29)	24 (17–28)	0.26
DMFT (SD)	18.9 (6.9)	20.9 (4.6)	17.6 (5.8)	0.29
PCR (SD)	31.6 (12.8)	44.5 (17.8)	33.4 (16.5)	0.1
Salivary flow rate (mL/min)				
Mean (SD)	1.37 (0.60)	1.04 (0.67)	1.03 (0.51)	0.19
≤1.0 mL/min (%)	33.3	53.3	52.9	0.28
>1.0 mL/min (%)	66.7	46.7	47.1	0.35
Salivary bacterial count				
Streptococci (10^7^/mL)	6.69	8.83	3.9	0.06
Mutans streptococci				
*S. mutans* (10^4^/mL)	2.7	6.1	3.6	0.11
*S. sobrinus* (10^5^/mL)	0.4	10.3	1.7	0.18
Lactobacillus (10^7^/mL)	1	13.8	5.3	0.25

**Table 3:** Distribution of the tooth types included in this study.

Tooth	Caredyne	Fuji VII	NaF
Maxilla			
Incisor	4 (2)	2 (2)	8 (6)
Canine	2(2)	2 (1)	1 (1)
Premolar	5 (4)	3 (1)	1
Molar	3 (3)	1	3 (2)
Mandible			
Incisor	0	2 (2)	2 (1)
Canine	1	6 (3)	0
Premolar	3 (1)	6 (4)	3 (3)
Molar	0	0	0

Parentheses indicate values for the teeth on the right side.

**Table 4:** Baseline characteristics of the teeth.

	Caredyne	Fuji VII	NaF
Color			
Pale yellow			1
Light brown	10	9	8
Dark brown	6	6	4
Black	2	7	5
Hardness			
Soft	2	5	1
Leathery	16	17	17

## Data Availability

The data presented in this study are available upon reasonable request from the corresponding author. The data cannot be publicly accessed owing to disagreements among the study participants.

## Supplementary Materials

The following supporting information can be downloaded at https://www.mdpi.com/article/10.3390/medicina60010150/s1: Table S1: Characteristics of teeth in which material fall-out was observed.
